# Supplementary material for: Relationships Among Origin, Genotype, and Oenological Traits of Brettanomyces Yeasts
Source: Int J Mol Sci. 2024 Nov 2;25(21):11781. doi: 10.3390/ijms252111781 (PMC11547160; doi:10.3390/ijms252111781)
Supplement: Supplementary file 1 [file ijms-25-11781-s001.zip › ijms-3282803-supplementary.pptx]

## Slide 1
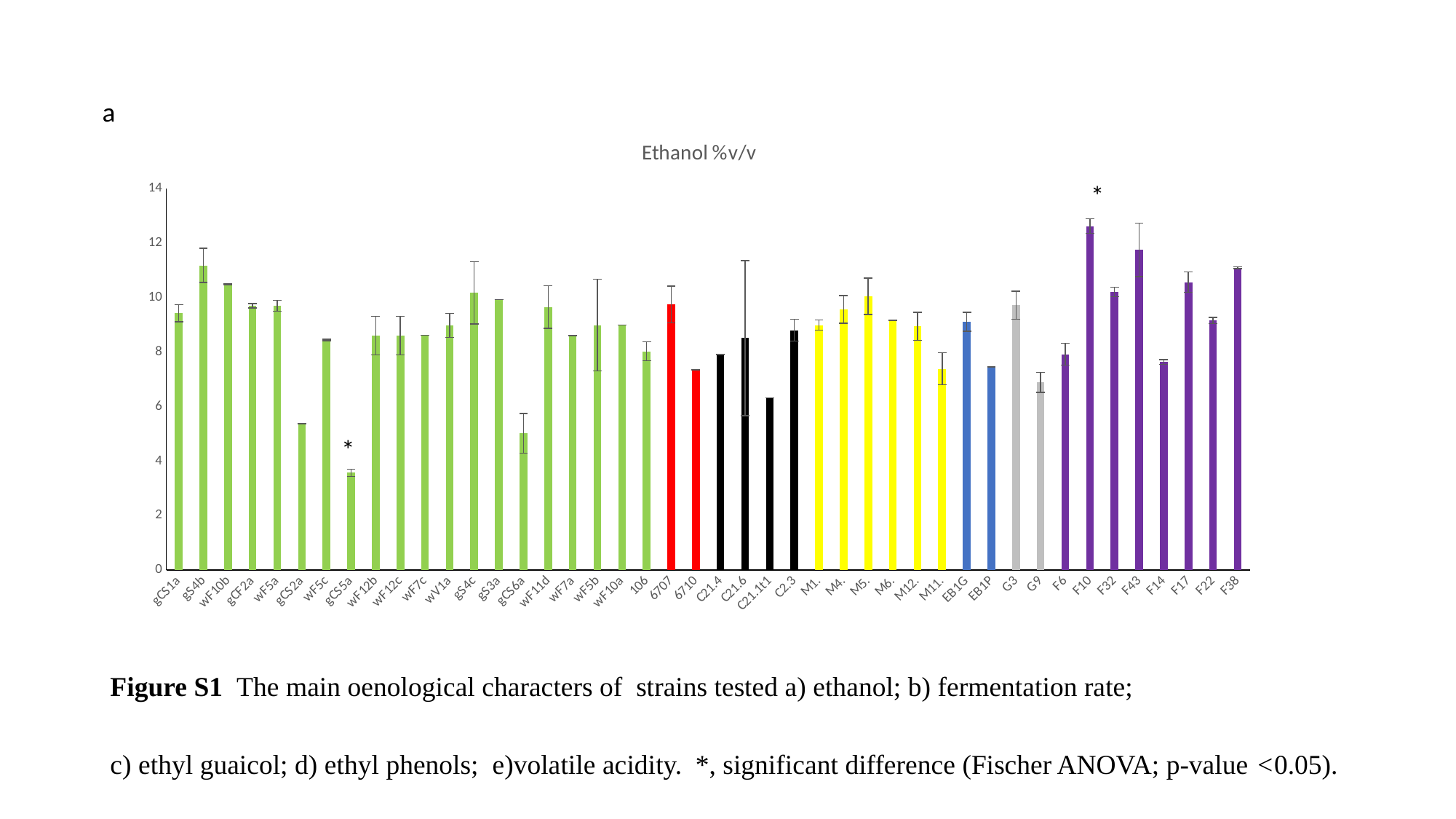

a
### Chart: Ethanol %v/v
| Category | ETANOLO TEORICO |
|---|---|
| gCS1a | 9.425099999999983 |
| gS4b | 11.1784 |
| wF10b | 10.473700000000001 |
| gCF2a | 9.70099999999999 |
| wF5a | 9.70099999999999 |
| gCS2a | 5.375600000000018 |
| wF5c | 8.4445 |
| gCS5a | 3.5688999999999904 |
| wF12b | 8.606300000000008 |
| wF12c | 8.606300000000008 |
| wF7c | 8.62 |
| wV1a | 8.9801 |
| gS4c | 10.172699999999981 |
| gS3a | 9.93 |
| gCS6a | 5.019600000000009 |
| wF11d | 9.647600000000008 |
| wF7a | 8.6 |
| wF5b | 8.989 |
| wF10a | 8.99 |
| 106 | 8.027799999999981 |
| 6707 | 9.74550000000001 |
| 6710 | 7.3514 |
| C21.4 | 7.903199999999991 |
| C21.6 | 8.51 |
| C21.1t1 | 6.319000000000019 |
| C2.3 | 8.80210000000001 |
| M1. | 8.989000000000019 |
| M4. | 9.567500000000017 |
| M5. | 10.039200000000017 |
| M6. | 9.15809999999999 |
| M12. | 8.944500000000035 |
| M11. | 7.386999999999983 |
| EB1G | 9.11 |
| EB1P | 7.4582 |
| G3 | 9.717199999999991 |
| G9 | 6.89 |
| F6 | 7.9209999999999825 |
| F10 | 12.6202 |
| F32 | 10.2067 |
| F43 | 11.747999999999966 |
| F14 | 7.63620000000002 |
| F17 | 10.56429999999995 |
| F22 | 9.15809999999999 |
| F38 | 11.091000000000001 |*
*
Figure S1 The main oenological characters of strains tested a) ethanol; b) fermentation rate;
c) ethyl guaicol; d) ethyl phenols; e)volatile acidity. *, significant difference (Fischer ANOVA; p-value <0.05).

## Slide 2
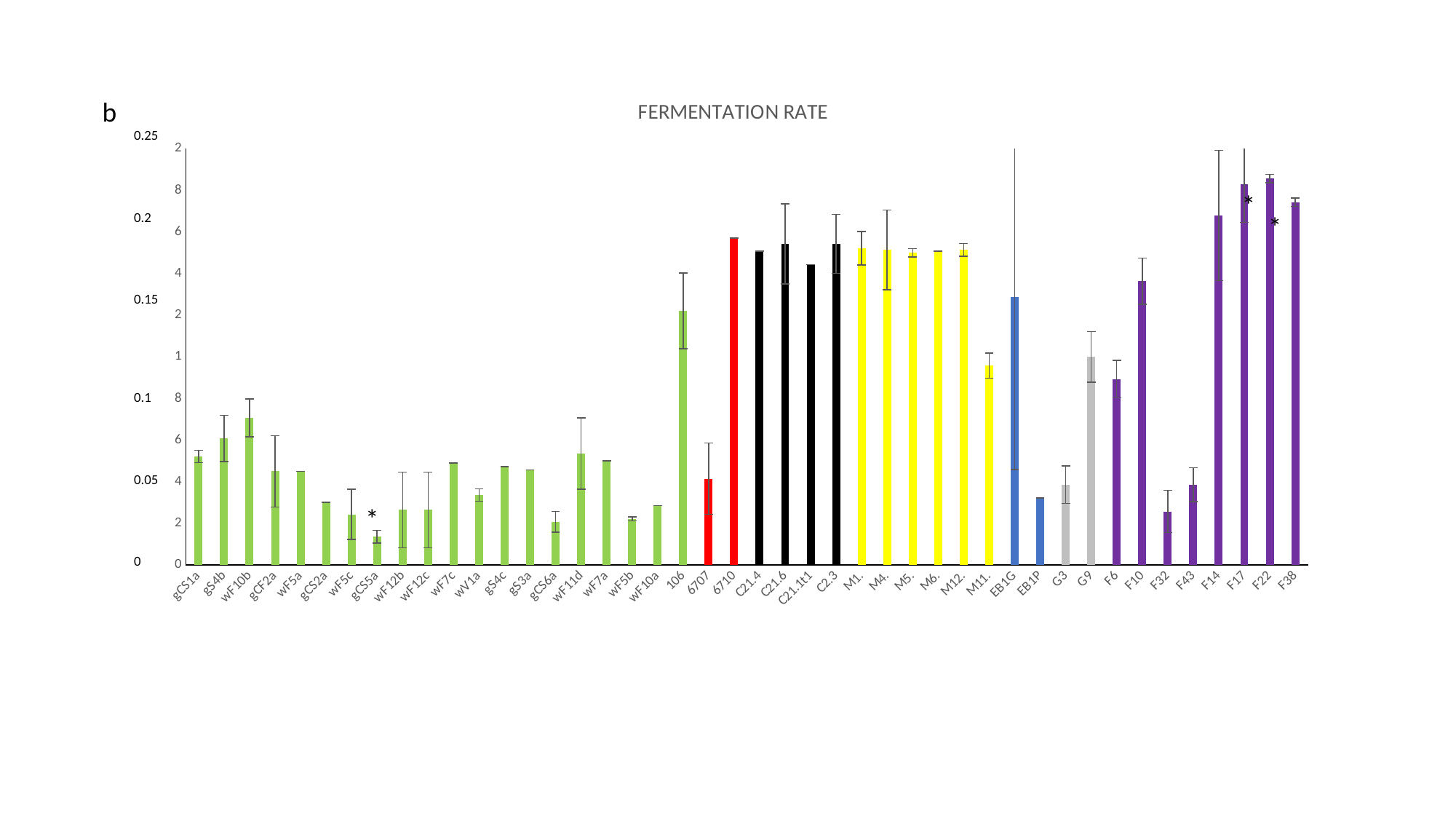

### Chart: FERMENTATION RATE
| Category | |
|---|---|
| gCS1a | 0.05214285714285635 |
| gS4b | 0.06071428571428736 |
| wF10b | 0.07071428571428635 |
| gCF2a | 0.04500000000000172 |
| wF5a | 0.04500000000000172 |
| gCS2a | 0.030000000000001144 |
| wF5c | 0.0242857142857125 |
| gCS5a | 0.013571428571428394 |
| wF12b | 0.026428571428571787 |
| wF12c | 0.026428571428571787 |
| wF7c | 0.049 |
| wV1a | 0.0335714285714285 |
| gS4c | 0.047142857142854856 |
| gS3a | 0.045714285714284715 |
| gCS6a | 0.020714285714287215 |
| wF11d | 0.053571428571428575 |
| wF7a | 0.049999999999999996 |
| wF5b | 0.022142857142855285 |
| wF10a | 0.028571428571428574 |
| 106 | 0.12214285714285571 |
| 6707 | 0.041428571428574354 |
| 6710 | 0.15714285714285572 |
| C21.4 | 0.150714285714285 |
| C21.6 | 0.1542857142857143 |
| C21.1t1 | 0.14428571428571685 |
| C2.3 | 0.15428571428571358 |
| M1. | 0.15214285714285708 |
| M4. | 0.15142857142857144 |
| M5. | 0.15 |
| M6. | 0.1507142857142843 |
| M12. | 0.15142857142857144 |
| M11. | 0.09571428571428643 |
| EB1G | 0.1285714285714286 |
| EB1P | 0.03214285714285715 |
| G3 | 0.038571428571428576 |
| G9 | 0.09999999999999999 |
| F6 | 0.08928571428571222 |
| F10 | 0.13642857142857112 |
| F32 | 0.025714285714284642 |
| F43 | 0.038571428571426 |
| F14 | 0.16785714285714215 |
| F17 | 0.18285714285714286 |
| F22 | 0.185714285714285 |
| F38 | 0.17428571428571643 |0.25
0.2
0.15
0.1
0.05
0
*
*
*
b

## Slide 3
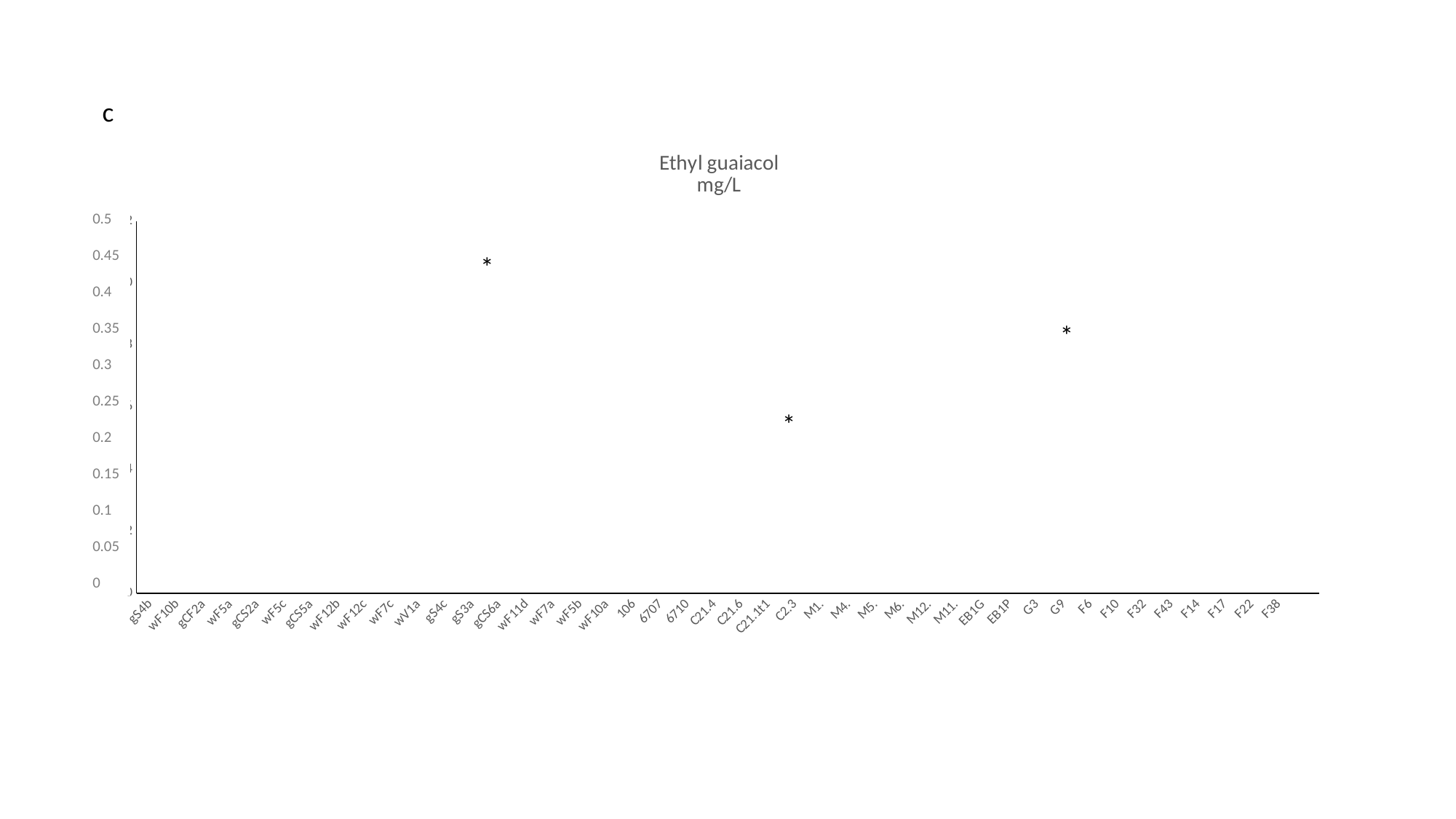

c
### Chart: Ethyl guaiacol
mg/L
| Category | |
|---|---|
| gS4b | 0.29700000000000004 |
| wF10b | 0.162 |
| gCF2a | 0.178 |
| wF5a | 0.178 |
| gCS2a | 0.244 |
| wF5c | 0.277 |
| gCS5a | 0.254 |
| wF12b | 0.146 |
| wF12c | 0.146 |
| wF7c | 0.175 |
| wV1a | 0.189 |
| gS4c | 0.157 |
| gS3a | 0.415 |
| gCS6a | 0.064 |
| wF11d | 0.2175 |
| wF7a | 0.177 |
| wF5b | 0.1945 |
| wF10a | 0.176 |
| 106 | 0.1525 |
| 6707 | 0.1975 |
| 6710 | 0.216 |
| C21.4 | 0.1115 |
| C21.6 | 0.06 |
| C21.1t1 | 0.1845 |
| C2.3 | 0.153 |
| M1. | 0.224 |
| M4. | 0.126 |
| M5. | 0.172 |
| M6. | 0.20750000000000002 |
| M12. | 0.137 |
| M11. | 0.1475 |
| EB1G | 0.163 |
| EB1P | 0.4215 |
| G3 | 0.307 |
| G9 | 0.16 |
| F6 | 0.217 |
| F10 | 0.267 |
| F32 | 0.1385 |
| F43 | 0.1935 |
| F14 | 0.14750000000000002 |
| F17 | 0.16749999999999998 |
| F22 | 0.22449999999999998 |
| F38 | 0.0945 |*
*
*
0.5
0.45
0.4
0.35
0.3
0.25
0.2
0.15
0.1
0.05
0

## Slide 4
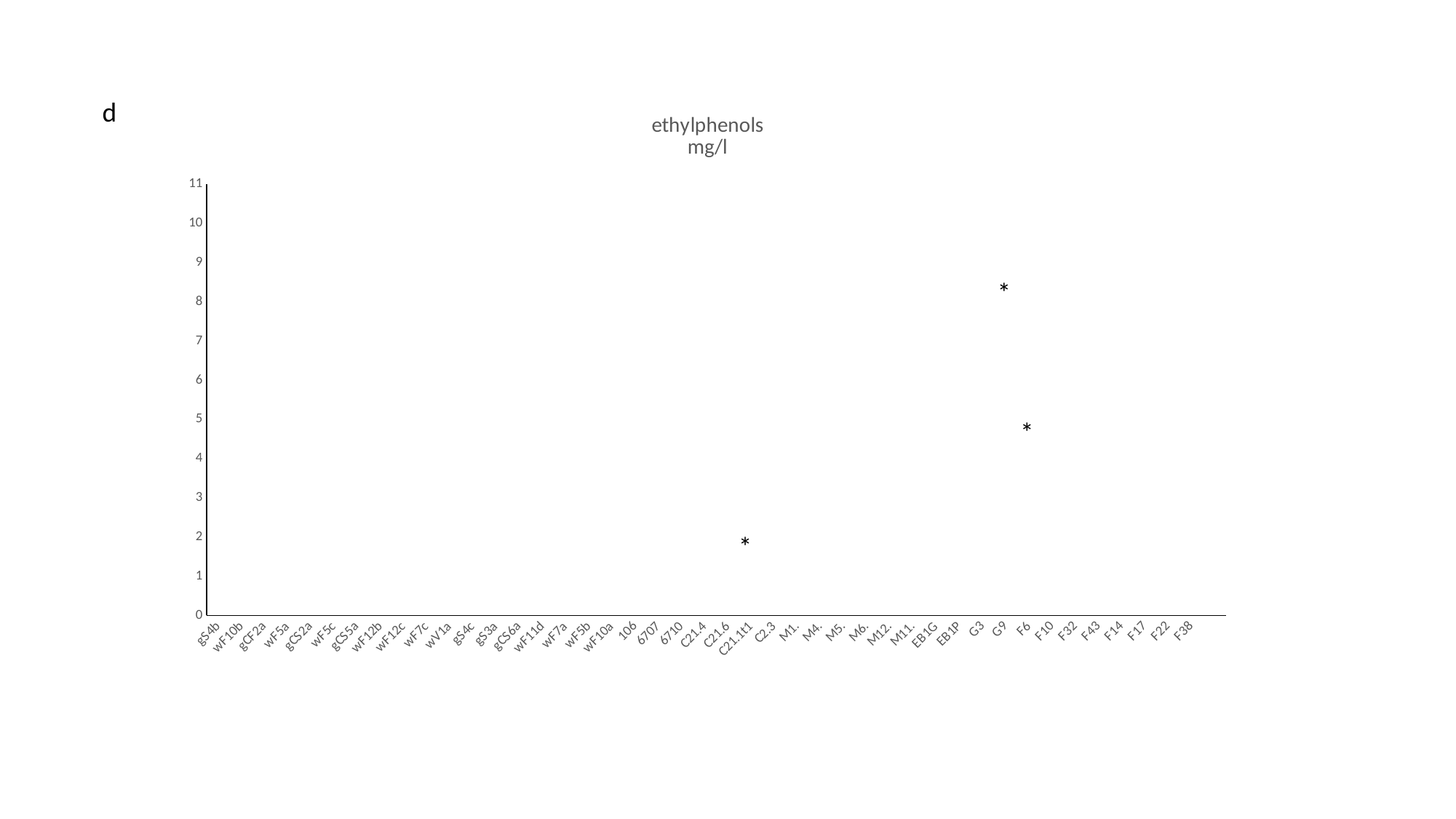

d
### Chart: ethylphenols
mg/l
| Category | |
|---|---|
| gS4b | 1.1895 |
| wF10b | 0.9844999999999999 |
| gCF2a | 0.914 |
| wF5a | 0.914 |
| gCS2a | 1.236 |
| wF5c | 1.2375 |
| gCS5a | 0.9185000000000001 |
| wF12b | 0.7535000000000001 |
| wF12c | 0.7535000000000001 |
| wF7c | 0.934 |
| wV1a | 0.8605 |
| gS4c | 0.798 |
| gS3a | 1.564 |
| gCS6a | 0.609 |
| wF11d | 0.8955 |
| wF7a | 0.874 |
| wF5b | 0.9219999999999999 |
| wF10a | 1.223 |
| 106 | 0.5800000000000001 |
| 6707 | 0.7645 |
| 6710 | 0.932 |
| C21.4 | 0.8554999999999999 |
| C21.6 | 0.358 |
| C21.1t1 | 0.7655000000000001 |
| C2.3 | 0.7105 |
| M1. | 0.964 |
| M4. | 0.679 |
| M5. | 0.711 |
| M6. | 0.7909999999999999 |
| M12. | 0.831 |
| M11. | 0.644 |
| EB1G | 0.906 |
| EB1P | 2.2195 |
| G3 | 2.1215 |
| G9 | 0.881 |
| F6 | 0.7505 |
| F10 | 1.1495 |
| F32 | 0.6485000000000001 |
| F43 | 0.6675 |
| F14 | 0.756 |
| F17 | 0.876 |
| F22 | 0.9445 |
| F38 | 0.506 |*
*
*

## Slide 5
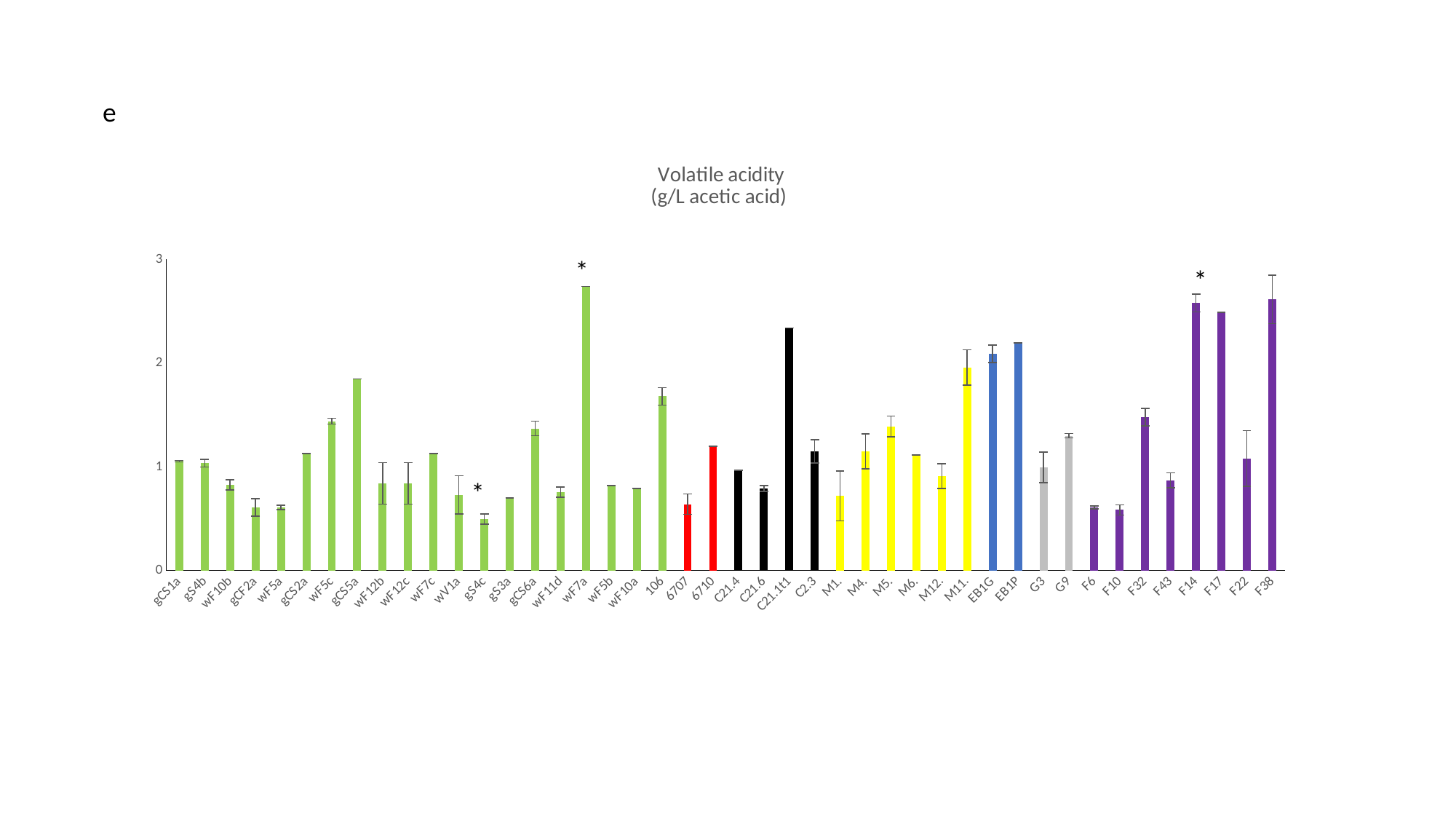

e
### Chart: Volatile acidity
(g/L acetic acid)
| Category | |
|---|---|
| gCS1a | 1.0545 |
| gS4b | 1.0350000000000001 |
| wF10b | 0.825 |
| gCF2a | 0.6100000000000001 |
| wF5a | 0.6100000000000001 |
| gCS2a | 1.13 |
| wF5c | 1.44 |
| gCS5a | 1.85 |
| wF12b | 0.84 |
| wF12c | 0.84 |
| wF7c | 1.13 |
| wV1a | 0.73 |
| gS4c | 0.495 |
| gS3a | 0.7 |
| gCS6a | 1.37 |
| wF11d | 0.755 |
| wF7a | 2.74 |
| wF5b | 0.82 |
| wF10a | 0.79 |
| 106 | 1.6800000000000002 |
| 6707 | 0.6399999999999999 |
| 6710 | 1.2 |
| C21.4 | 0.966 |
| C21.6 | 0.79 |
| C21.1t1 | 2.34 |
| C2.3 | 1.15 |
| M1. | 0.72 |
| M4. | 1.15 |
| M5. | 1.39 |
| M6. | 1.115 |
| M12. | 0.91 |
| M11. | 1.96 |
| EB1G | 2.09 |
| EB1P | 2.195 |
| G3 | 0.995 |
| G9 | 1.3 |
| F6 | 0.61 |
| F10 | 0.585 |
| F32 | 1.48 |
| F43 | 0.87 |
| F14 | 2.58 |
| F17 | 2.4935 |
| F22 | 1.08 |
| F38 | 2.615 |*
*
*

## Slide 6
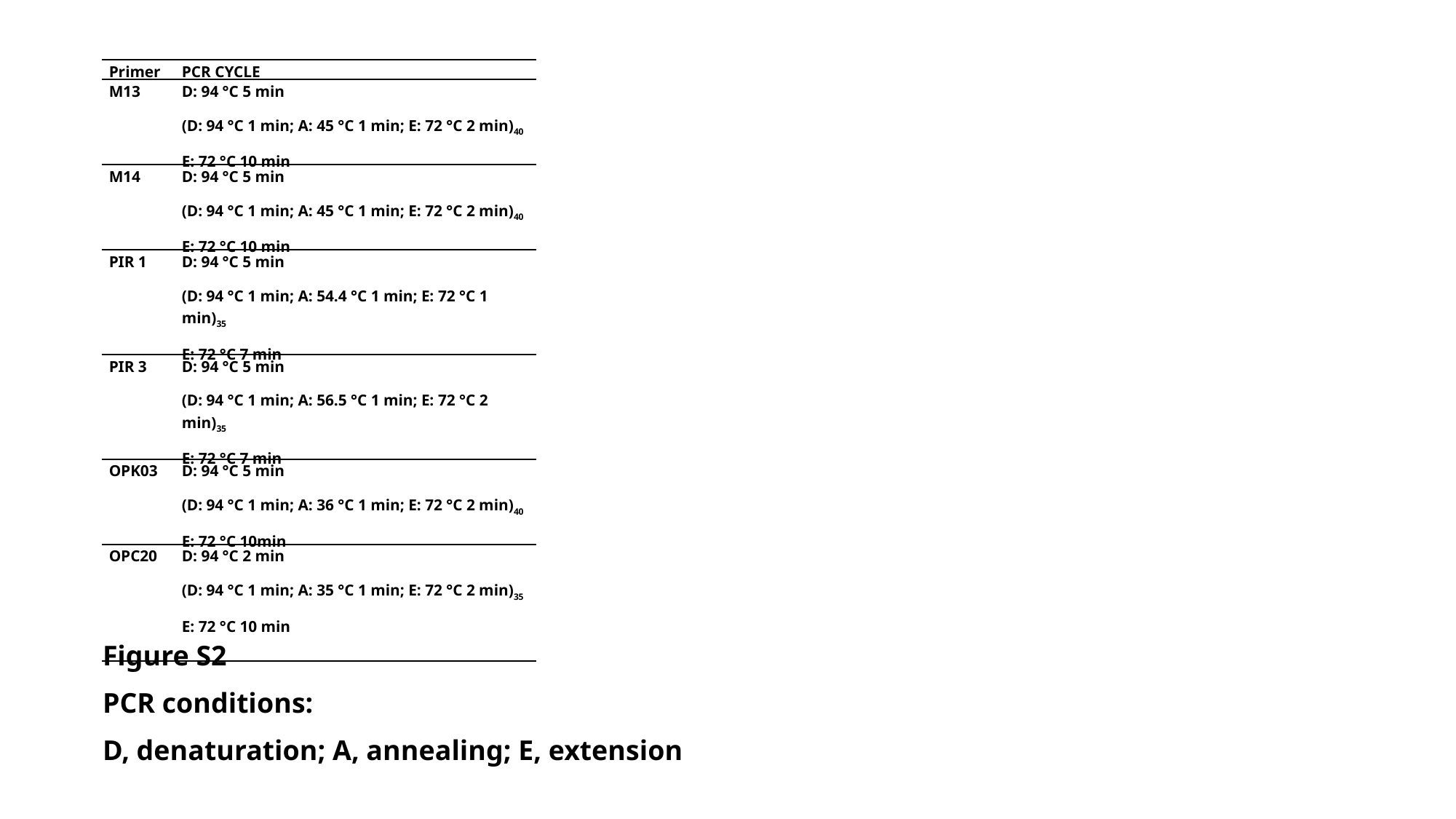

| Primer | PCR CYCLE |
| --- | --- |
| M13 | D: 94 °C 5 min (D: 94 °C 1 min; A: 45 °C 1 min; E: 72 °C 2 min)40 E: 72 °C 10 min |
| M14 | D: 94 °C 5 min (D: 94 °C 1 min; A: 45 °C 1 min; E: 72 °C 2 min)40 E: 72 °C 10 min |
| PIR 1 | D: 94 °C 5 min (D: 94 °C 1 min; A: 54.4 °C 1 min; E: 72 °C 1 min)35 E: 72 °C 7 min |
| PIR 3 | D: 94 °C 5 min (D: 94 °C 1 min; A: 56.5 °C 1 min; E: 72 °C 2 min)35 E: 72 °C 7 min |
| OPK03 | D: 94 °C 5 min (D: 94 °C 1 min; A: 36 °C 1 min; E: 72 °C 2 min)40 E: 72 °C 10min |
| OPC20 | D: 94 °C 2 min (D: 94 °C 1 min; A: 35 °C 1 min; E: 72 °C 2 min)35 E: 72 °C 10 min |
Figure S2
PCR conditions:
D, denaturation; A, annealing; E, extension

## Slide 7
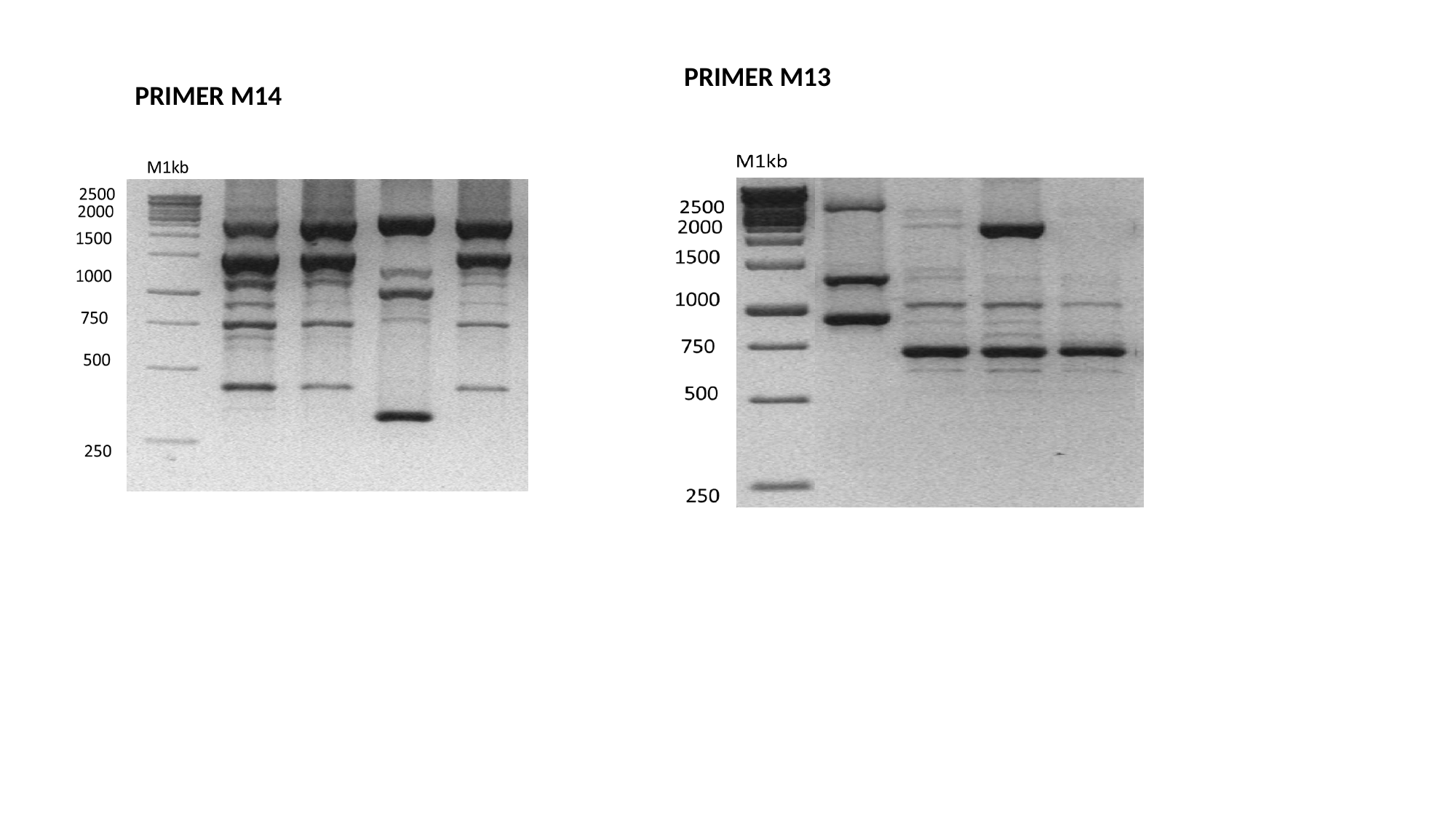

PRIMER M13
PRIMER M14

## Slide 8
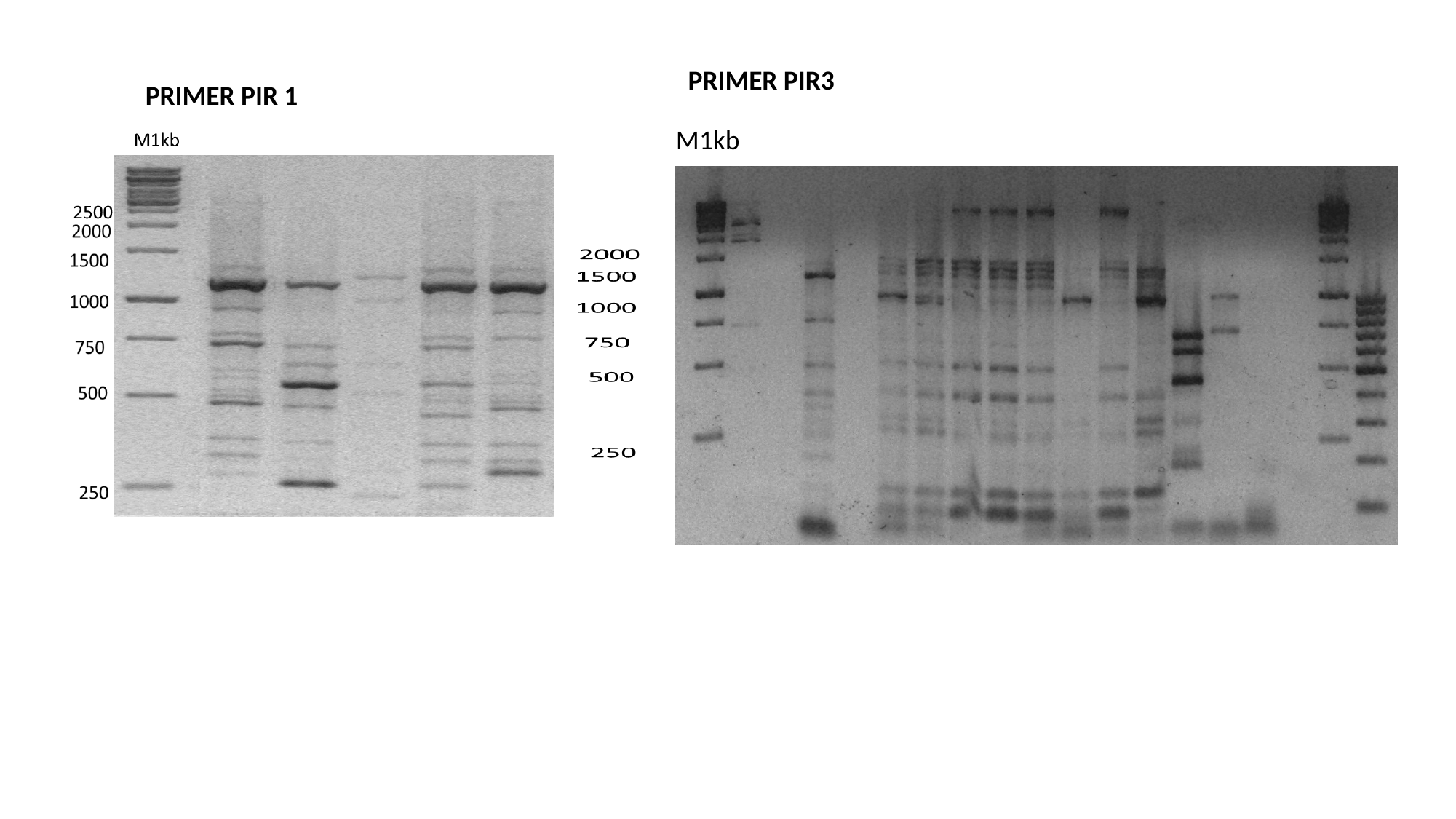

PRIMER PIR3
PRIMER PIR 1
M1kb

## Slide 9
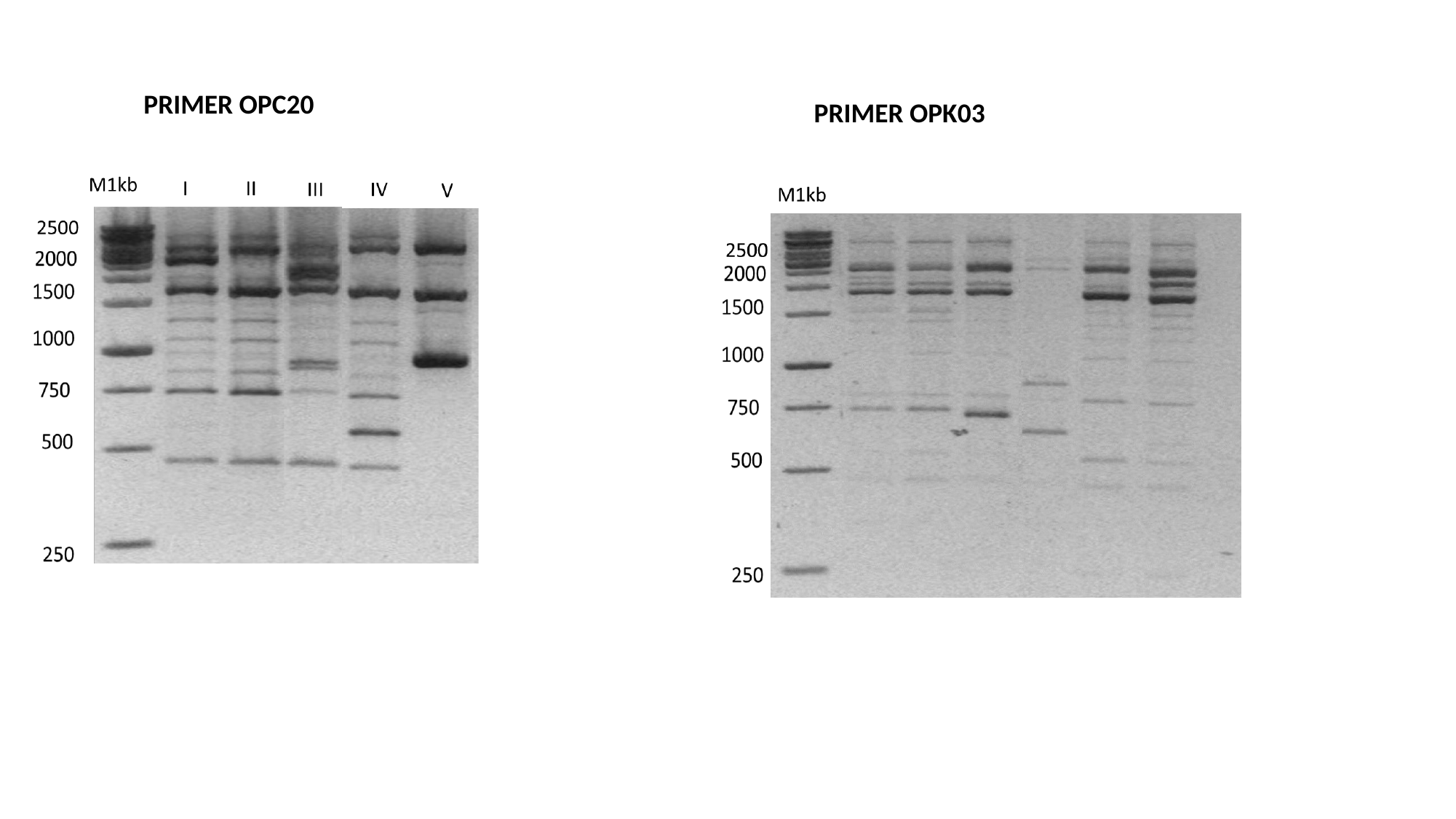

PRIMER OPC20
PRIMER OPK03
